# Analytical and Sample Preparation Techniques for the Determination of Food Colorants in Food Matrices

**DOI:** 10.3390/foods9010058

**Published:** 2020-01-07

**Authors:** Konstantina Ntrallou, Helen Gika, Emmanouil Tsochatzis

**Affiliations:** 1 Department of Chemical Engineering, Aristotle University of Thessaloniki, 54124 Thessaloniki, Greece; kondrallou@gmail.com; 2 Laboratory of Forensic Medicine & Toxicology, Department of Medicine, Aristotle University of Thessaloniki, 54124 Thessaloniki, Greece; gkikae@auth.gr; 3 BIOMIC AUTH Center for Interdisciplinary Research of the Aristotle University of Thessaloniki, Innovation Area of Thessaloniki, 57001 Thermi, Greece

**Keywords:** food colorants (synthetic, natural), food matrices, instrumental analysis, sample preparation

## Abstract

Color additives are widely used by the food industry to enhance the appearance, as well as the nutritional properties of a food product. However, some of these substances may pose a potential risk to human health, especially if they are consumed excessively and are regulated, giving great importance to their determination. Several matrix-dependent methods have been developed and applied to determine food colorants, by employing different analytical techniques along with appropriate sample preparation protocols. Major techniques applied for their determination are chromatography with spectophotometricdetectors and spectrophotometry, while sample preparation procedures greatly depend on the food matrix. In this review these methods are presented, covering the advancements of existing methodologies applied over the last decade.

## 1. Introduction

Codex Alimentarius gives a definition for food additives as “any substance that its intentional addition of which to a food aiming for a technological (including organoleptic) purpose in the manufacture, processing, preparation treatment, packing, packaging, transport or holding of such food results, or may be reasonably expected to result, in it or its by-products becoming a component of the food or otherwise affecting the characteristics of such foods” [1,2]. Carocho et al. highlighted that the definition given by the Codex Alimentarius does not include the term contaminants or substances added to food for maintaining or improving nutritional qualities [2].

In food technology, food colorants, of several types, are chemical substances that are added to food matrices, to enhance or sustain the sensory characteristics of the food product, which may be affected or lost during processing or storage, and in order to retain the desired color appearance [3,4,5]. These are classified based on several criteria: firstly, based on their origin in nature, nature-identical or, if synthetic, whether they are organic or inorganic. Another classification could be based on their solubility (e.g., soluble or insoluble) or covering ability (e.g., transparent or opaque), though an overlap may exist among one or more of these classifications. The most common and widely used classification is based on the distinction between soluble and insoluble color additives (colorants or pigments), which can be further categorized as natural or synthetic [4].

In addition, as described by Martins et al., there were several food additives that had been used extensively in the past but are no longer allowed, due to existing evidence of their side effects, toxicity in the medium- and long-term, as well as a high frequency of potential health incidents [6]. It is also important to note that, apart from synthetic food colorants, certain commercial additives of plant or animal origin have also been suspended [3,6,7,8].

It is clear that the analysis of trace amounts of food colorants is essential with the proper analytical techniques applied, with high specificity and selectivity. Ni et al. has reported that there is increasing interest in the monitoring of the concentration of synthetic food colorants in various products [9].

The analytical methods and sample preparation protocols presented hereafter cover the main techniques that have been applied over the last decade (2008 onwards).

## 2. Natural Food Colorants

Natural additives have been used since ancient times. In certain cases, they were used for the preservation of foodstuffs. Nowadays, most consumers seem to be in favor of the use of the natural, as opposed to the synthetic ones, which are considered by the food industry to be more efficient. In the meantime, there is also considerable interest in the overall reduction of food colorants to food products [4,5,10]. The classification of naturally derived colorants can become very complex because of the wide variety of innate properties of the coloring substances. They can be derived from a variety of sources in nature, and therefore, natural colorants also exhibit a wide variety of chemical compositions that affect properties, solubilities, and stabilities differently, and they can have different sources as plant-origin or animal-origin [10].

As reported by Carocho et al., there are benefits linked with the use of natural additives over their respective synthetic ones, which in certain cases present a greater potency over the synthetic ones. The latter in most cases present a single effect on the foodstuff in question. Nevertheless, natural additives are often produced using different methods, i.e., extraction from plants or produced by microorganisms, although there is a tendency to consider them safer than their respective synthetic additive. In general, toxicity is a factor that must be thoroughly assessed and evaluated, to ensure health and safety [2,5,10].

Synthetic colorants have a large span of application and are proportionally lower in cost, than their respective natural substances. However, natural colorants are gradually replacing the synthetic ones as they tend to be considered safer, while presenting higher color specificity, no side effects or related toxicity, and conferring health improving effects and functional benefits to the food itself [6,11,12]. A good example for this beneficial effect is the class of yeast-derived natural pigments (e.g., monascin; a yellow natural pigment). These present certain features, apart from food coloring, such as biological activity, reported potential anti-cancer, anti-inflammatory, anti-diabetic, and anti-cholesterolemic effects [6,13,14].

As reported by Martins et al., numerous references highlighted the effective and/or selective use of food colorants. Therefore, for the approved food colorants with an “E” code, individual Acceptable Daily Intakes (ADI) have been approved and established, expressed mostly as mass fractions (i.e., mg/kg per body weight (b.w), which can be used for specific purposes (i.e., colorants) in specific food products (i.e., biscuits, chocolates, cheeses etc.) [6].

Commonly, naturally occurring food colorants can be allocated in different sub-categories, namely anthocyanins, carotenoids, beet colorants, and phenolic compounds. In addition, annatto, carminic acid, and some curcuminoids have been studied, particularly curcumin. Finally, other colorants remain to be assessed and evaluated in order to be authorized with an “E” code.

Anthocyanins are a widely studied natural food colorants group, mainly obtained from flowers, fruits, leaves, and even whole plants with a color range that goes from red to purple and blue. Commercial anthocyanins, such as cyanidin 3-glucoside, pelargonidin 3-glucoside, and peonidin 3-glucoside have been used effectively [2,4,6].

Carotenoids are another cluster of naturally derived colorants with a renowned technological effect. They present coloring attributes along with certain bioactive as well as antioxidant properties and are being used extensively in the food industry as natural preservatives [4,6,7,10,15] apart from food colorants [7]. Their main source is extracts from plant roots, flowers, and leaves, as well as from algae, yeasts, and aquatic animals. This category mainly includes Lutein, astaxanthin, and lycopene [2,6], the most widely used carotenoids used with others such as crocin and crocetin, still under investigation [4,5,6].

Red-purple colorants derived from beets and beetroot (Beta vulgaris L.) root is the principal source of these natural colorants but also fruit of *Hylocereus polyrhizus* (Weber) Britton and Rose, *Opuntia ficus-indica* [L.] Miller, *Opuntia stricta* (Haw). Haw and *Rivina humilis* L. are also rich in these colorant substances, namely, the betacyanins and betalains, which are the most frequently studied and already authorized (E162). They are being used in various food products such as burgers, desserts, ice creams, jams, jellies, soups, sauces, sweets, drinks, dairy products, and yogurts [2,4,5,6].

Other natural food colorants are considered the phenolic compounds, where flavanones, flavones (4′,5,7-trihydroxyflavones), and flavonols (fisetin, myricetin, myricitin, quercetin, and rutin) have been studied. As reported by Martins et al., currently only the commercially available products are being used (i.e., myricetin and myricitrin from *Myrica cerifera* L. roots). Phenolic compounds do not yet have an approved “E” code nor an ADI value [6] with many still being studied and examined since their safety, stability, and spectrum of activity still remain unclear [6,16].

Another category of natural food colorant is the curcuminoids with the most widely known and used colorant in this group being curcumin (E100), usually isolated from Curcuma longa L. rhizomes.

Other natural used colorants are the annatto (E160b) group, as well as bixin and norbixin which are extracts from *Bixa orellana* L. seeds [2,4,5,6]. In addition, carminic acid (E120) with a yellow to red-orange food color is already largely used, either naturally occurring or of synthetic origin with an ADI of 5 mg/kg b.w [6] or crocin. Nevertheless, there are other food colorants under investigation, including c-phycocyanin (blue pigment isolated from Arthrospira platensis) and c-phycoerythrin (red-orange pigment from blue-green algae). Other naturally occurring pigments, which are commercially available, are being studied, such as geniposide, monascorubrin, and purple corn color [4,5,6].

## 3. Synthetic Food Colorants

Based on increasing demand, mainly from the consumer, for products that are more visually attractive, several synthetic food colorants have been developed for use in food production, to increase certain quality and organoleptic characteristics. However, it is reported that over time, most of the synthetic food colorants were excluded due to repeated side effects as well as to their short- and/or long-term toxicity and eventually to potential carcinogenic effects [3,6,11].

Thus, a change in consumer expectations has been reported, which is largely in favor of the natural colorants [6,17].

Apart from this, also from a regulatory point of view, there is increasing attention and interest related to the risk assessment of these colorants used in food products (i.e., azo-dyes). In case of the azo-dyes, a limiting factor for their use is their potential carcinogenicity, which occurs after their reduction to carcinogenic metabolites into the intestine [3,18,19]. These metabolites are produced in the human body, though their toxic effect depends on the ingested amount of the target substance/colorant [3,18,20]. However, it is reported that regular evaluation and assessment of potential toxicity of food colorants by regulatory authorities is necessary [3,18,21].

## 4. Toxicological Aspects and Regulatory Framework

Based on various scientific findings, several toxicity effects, have been reported including behavioral effects on children, effects on the respiratory system, connection with allergies, development of attention deficit hyperactivity disorder (ADHD) in children, or neuro-developmental effects at the No-Adverse Effect Limit levels [3,18,21]. In any case, further investigation to assess the potential associated risks of these compounds is needed [3,4,5,6,7,8,9,11,14,18].

**Figure 1:**
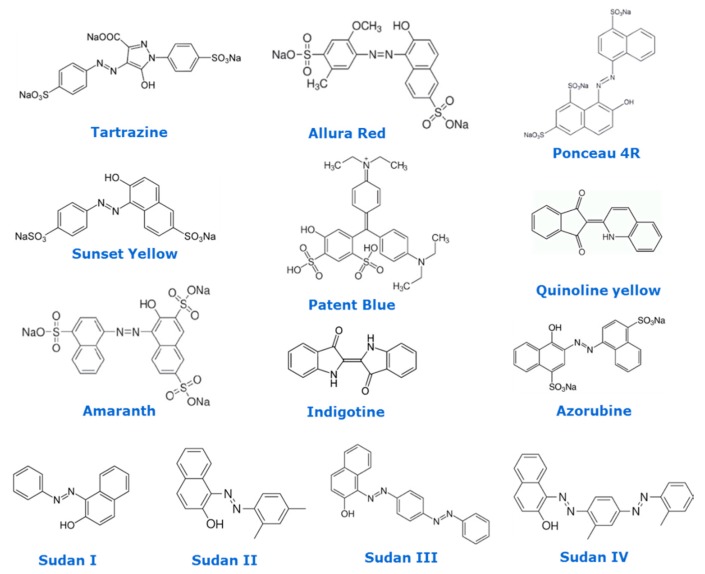
Chemical structures of selected regulated food colorants.

Several groups have indicated the toxic effect of some of groups of these substances. As an example, Mpountoukas et al. have tested the food colorants amaranth, erythrosine, and tartrazine by in vitro experiments, and they concluded there was an in vitro toxic effect on human lymphocytes as they bound to DNA [22]. Many other studies have shown the chemical property of synthetic colorants, namely, Tartrazine [23], azorubine [17,24,25], Allura Red [17,26,27] Sunset Yellow, Quinoline Yellow [17], and Patent Blue [28], to bind to human serum albumin (HSA). Masone and Chanforan compared binding affinities of artificial colorants to human serum albumin (HSA), exhibiting more affinity to HSA than to their natural equivalents’ colorants and interacting with its functions. The results supported the hypothesis of their potential risk to human health [17]. Finally, there are dyes, which are rather inexpensive, and which have been used in the food industry, such as Sudan I–IV, which are classified as both a toxic and carcinogenic [24,25,26,27,28,29,30,31]. In Figure 1, basic structures of colorants used in the food industry some of them with toxicological concern (Sudan I–IV) are presented.

The main regulatory authorities, EFSA in Europe and the US Food and Drug Administration (FDA) in the United States, are responsible for the evaluation and assessment of food products to enhance and promote health safety [2,4,5]. The European Union, set a re-evaluation program of food additives, including food colorants, to be performed by EFSA by 2020, based on the EU Regulation 257/2010. This re-evaluation program was set in order to assess the safety of all authorized food additives in the European Union before 20 January 2009 [32].

The regulatory framework in Europe, in brief, contains the authorization procedure in Regulation (EU) No. 1331/2008, the rules on food additives with a list of approved color additives and their conditions of use in Regulations (EU) No 1333/2008 and 1129/2011, the specifications for food additives in Regulation (EU) 231/2012, and finally for labelling in Regulations (EU) No. 1169/2011 and 1333/2008. Respectively, in the United States, the color additives are included in Title 21 CFR Part 70, listing food additives (exempt from certification, including specifications and conditions of use) in Title 21 CFR Part 73, and certification of donor additives in Title 21 CFR Part 80 [4,5,10,33].

However, despite the existence of different regulatory frameworks, the overall approach follows similar steps, which are based on well-established risk assessment procedures [33].

Authorization for the use of food colorants in the production of food products is subject to a number of toxicity tests, in order to define and evaluate acute, sub-chronic and chronic toxicity, hepatotoxicity, carcinogenicity, mutagenicity, teratotoxicity, genotoxicity, reproductive toxicity, accumulation in the body, bioenergy effects, and immunotoxicity [3,4,5,6,7,8,9,11,14,18].

## 5. Analytical Methodologies for the Determination of Food Colorants

### 5.1. Analytical Techniques in the Use of Natural Food Colorants Determinations

The available bibliography concerning the methods of analysis for the natural colorants is limited, compared to that for the synthetic ones, and it is exclusively oriented to their determination in the different naturally deriving products.

All the relative information concerning analytical methods for natural colorants, including tested matrices, analytical technology, type of detection and settings, analytical columns if used, elution parameters, mobile phases, injection volumes, and analytical figures of merit (LOD, LOQ), have been reviewed and are summarized in Table 1.

It can be concluded from Table 1 that evaluation of methods’ performance criteria was not within the aims of the above-mentioned reports, as they were focusing in activity, bioavailability, processing impact, and adulteration. Thus, no analytical figures of merit are reported in these papers.

**Figure 2:**
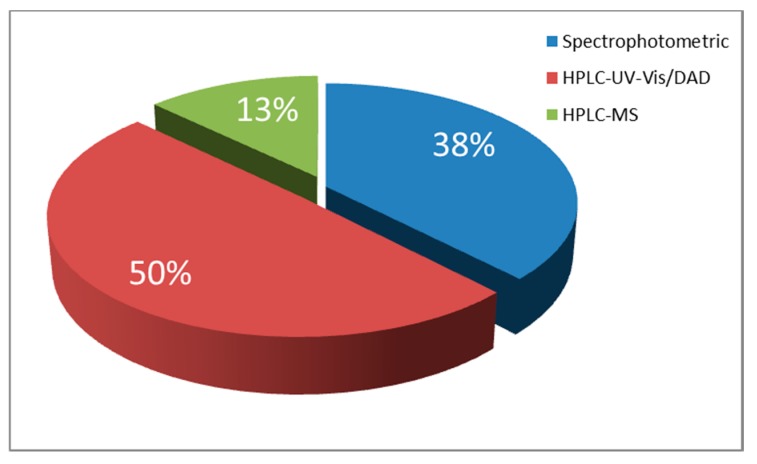
Distribution of techniques used for the analysis of natural food colorants.

From Table 1 and Figure 2 it could be perceived that the predominant technique is HPLC combined with spectrophotometric (UV-Vis) or Diode Array (DAD) detectors, followed by HPLC by MS/MS. Spectrophotometric UV-Vis methods seem also to be preferred by the researchers in this field as they show low instrument cost and do not involve expert skill. However, it should be considered that the individual features of the spectra obtained for single colors are highly dependent on the pH-adjustment of the solution or the mobile phase, using proper acid or alkali. The pH adjustment certainly affects maximum absorption wavelength, where shifts and intensities based on the different pH can be observed. Although sample preparation is much less demanding in comparison to the LC methods, these techniques present a significant disadvantage, which is the lack of ability to analyze simultaneously a bigger number of food colorants.

### 5.2. Sample Preparation for Natural Colorant Analysis

Several sample preparation protocols are reported in the literature by applying various techniques. The applied protocol is strongly dependent by the type and nature of the food sample. Below in Table 2, a short description of the sample preparation protocols is given, along with their application for the clean-up of food samples, for the quantification of natural food colorants. A hydrolysis step with a deprotonation step (ethanol, HCl solution) is being reported depending on the food matrix, including dilution methods and SFE with supercritical CO_2_.

### 5.3. Analytical Techniques in the Use of Synthetic Food Colorants Determinations

The need to determine synthetic colorants in food matrices originating from their known toxicity, renders the analytical task even more challenging as food matrices are ordinarily very complex. Various analytical techniques are used to determine synthetic food colorants in food samples, including spectrophotometry, thin layer chromatography, capillary electrophoresis, high performance liquid chromatography and mass spectrometry (MS).

Certain chemical properties and characteristics of the substances/colorants that influence their separation, such as hydrophilicity/hydrophobicity, existence of acidic or alkaline groups should to be taken into account. Using a Reversed Phase (RP) liquid chromatography separation, more polar compounds are eluting first followed by the less polar. However, their chromatographic separation is normally performed at neutral pH (ca. 7), and thus, any presence of acidic or alkaline groups could affect the elution sequence.

Ordinarily, organic solvents such as methanol, acetonitrile, or their mixture are used for analysis by HPLC. The addition of acetonitrile improves significantly chromatographic peaks’ shape (i.e., asymmetry). Nevertheless, the addition of an inorganic electrolyte as a chemical modifier to the mobile phase can be considered as important in order to advance the separation of all the ionizable species [12,28,37,49].

Food colorants are compounds that absorb exceedingly in the visible region. Thus, spectrophotometry is sufficient and appropriate for their quantitative analysis. It is generally preferred as a quite straightforward technique, with respective low instrumental cost (i.e., compared to MS/MS). However, in several cases, its main drawback is the lack of specificity, as in case of mixtures of absorbing species. A solution to overcome the problem of specificity is the application of mass spectrometry (MS). In this case, all spectral interventions or interferences, presented on UV–Vis/DAD detectors, are overpassed. High analytical sensitivity could succeed, even in more difficult food matrices, though after proper clean-up. In addition, tandem MS technique could provide structural information based on the molecular mass/ion and the respective fragmentation pattern. Regarding the ionization mode, in most cases, for synthetic colorants, the electro spray ionization (ESI) is preferred because synthetic food colorants are polar molecules, and their ionization efficiency depends on the existence of matrix interferences, present in sample or in the mobile phase. In general, negative mode (ESI-) is more effective, though in other non-regulated substances (i.e., Sudan I-IV) the positive ionization is preferred. During the MS/MS analysis, chemical modifiers (i.e., HCOONH_4_ or CH_3_COONH_4_) are added to the mobile phases, in order to improve and facilitate the better ionization of each target analyte.

Capillary electrophoresis follows in frequency of use the HPLC-DAD/UV-Vis or MS/MS techniques, applied for the quantification of food colorants. These methods present good separation of both small and large molecules, using high voltages. Other reported techniques are FIA (Flow Injection Analysis) and TLC (Thin Layer Chromatography). These could be considered as relatively simple analytical techniques, even for quantification, though in certain cases they could lack specificity and could be affected by matrix interferences.

For synthetic food colorants, all the respective references containing details about the tested matrices, analytical techniques, detection and settings, analytical columns if used, elution, mobile phases, injection volumes, and figures of merit (LOD, LOQ) are presented below in Table 3.

**Figure 3:**
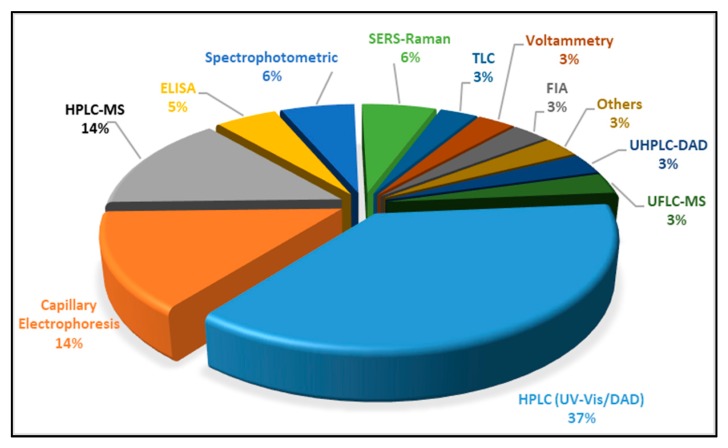
Distribution of techniques for the analysis of synthetic food colorants.

As it could be extrapolated from Table 3, a significant number of LC-MS, LC-MS/MS or LC-UV/Vis methods are available, which are dedicated to simultaneous detection of either a significant or limited number of artificial colorants (whether authorized or delisted), even including illegal Sudan-type dyes. In addition, to Table 3, Figure 3 gives the percentage distribution of the analytical techniques, regarding the analysis of synthetic food colorants. It could be easily concluded that HPLC/U(H)PLC is the most frequently applied technique, followed by capillary electrophoresis and enzyme-linked immunosorbent assay (ELISA) as well as other residual methods. In the case of ELISA, it needs to be highlighted that it cannot be applied for a group of substances/food colorants but only for standalone substances, for which the monoclonal antibodies have been developed.

The applied analytical techniques are followed by proper detection approaches. In this framework, simple detector UV-Vis/DAD is mostly applied, followed by MS/MS detectors, UV-Vis spectrometry, and electrochemical detection. The UV-Vis/DAD detection wavelengths depend on the analyte color (i.e., blue, yellow, red) set in any case in the maximum absorbance.

Regarding the MS, listed and EU-approved food colorants could be analyzed in the negative ionization, while for other substances (i.e., Sudan I-IV) positive ionization is applied.

From observation among the available methods of analysis (Table 3 and Figure 3), it could be concluded that traditional TLC methods require a significant sample preparation step and a time-consuming analytical procedure. On the other hand, the HPLC methods need longer analysis time, compared to the respective LC-MS/MS methods, in order to obtain good separation for the same number of analytes [87,88,89].

As reported recently by Periat et al., full-scan screening methods using HR-MS (High Resolution Mass Spectrometry) have proven to be an alternative to triple quadrupole methods as they could maximize the number of control and analyzed target colorants. Main advantages of the HR-MS can be the reduced sample preparation and the combined targeted analysis with untargeted screening of food colorants with high MS resolving power. Quadropole Time-of-Flight (QTOF) used by Li et al. and by Periat et al. for the detection and identification of coloring compounds in spices provided not only mass accuracy but also MS/MS spectra information and thus increased selectivity. A drawback of the approach could be the high cost of the instrumentation [85,86]. As reported by Li et al., HR-MS accurate mass measurements can detect a large number of target analytes, avoiding isobaric interferences in complex samples [89]. A combination of an ESI (or APCI) ionization with an anion trap analyzer linked to a TOF mass analyzer (ESI/APCI-IT-TOF/MS) provides simultaneously multi tandem MS (up to MS^2^) with respective mass accuracy. Currently, there is an increasing interest on the fragmentation mechanism of synthetic food dyes; use of ESI-IT-TOF/MS^n^ in positive as well as in negative ionization modes [87,88,89] has been increased.

### 5.4. Sample Preparation for the Determination of Synthetic Colorants in Foods

Currently, there is no generally accepted/standard method for synthetic colorant extraction in laboratories. Nevertheless, most extraction procedures follow a common approach, which normally involves firstly the release of desired analytes from their matrices, followed afterwards by removal of extraneous matter/interferences by applying an efficient extraction protocol (i.e., solid–liquid or liquid–liquid extraction) [90].

The applied sample preparation protocols are strongly dependent on the type and nature of the food sample. A short description of the sample preparation protocols, along with their application to the clean-up of food samples, for the analysis of synthetic food colorants is given in Table 4.

Membrane filtration involves the permeation of the analyte through a thin layer of material. Explicitly, in case of beverages, when filtration is involved, a degassing step needs to be done in advance, in order to remove CO_2_ [90].

Solid phase extraction (SPE) is one of the most commonly used techniques in determination of food colorants, presenting certain advantages such as simplicity. Polyamide resin used for SPE cleanup retains polar compounds with chemical groups that can be protonated. In acidic pH, during SPE, the colorants are adsorbed to the polyamide stationary phase mainly by Van der Waals interactions. Other hydrophilic substances can mask SPE interaction sites by reducing their binding power for the colorants and consequently reducing the capacity of the cartridges. Some substances, such as amaranth, are strongly retained by SPE cartridges, and the ammonia solution used for elution could be insufficient for its release (low recoveries).

Dispersive solid phase extraction (d-SPE) analysis is a simple sample preparation methodology that is suitable for a wide variety of food and agricultural products, as is also QuEChERS, introduced for pesticides from Anastassiades et al. [91]. In case of synthetic colorants, a modified QuEChERS method has been reported (magnetic-dSPE) using cross-linking magnetic polymer (NH_2_-LDC-MP) containing less hydrophilic amino groups and more lipophilic styrene monomer for cleaning up the synthetic food colorants from wine and soft drinks [53].

Liquid–liquid extraction (LLE) deals with the separation of substances based on their relative solubility in two different immiscible liquids. Common solvents for the extraction of synthetic food colorants from food matrices are water, ethanol, methanol, isopropyl alcohol, ammoniac ethanol, ethyl acetate, ammonia, cyclohexane, and tetra-n-butyl ammonium phosphate. Wu et al. has also reported an extraction method based on Ionic liquid dispersive liquid phase microextraction using the ionic liquid (1-Octyl-3-methylimidazolium tetrafluoroborate ((C_8_MIM)(BF_4_))) [81].

In the literature, a limited number of protocols exists dealing with other types of extraction methods for synthetic food colorants, such as MAE and Ultrasound Assisted Extraction (UAE). These kinds of extractions require special instrumentation and most probably can be beneficial for a laboratory, as extractions with organic solvents are characterized by consumption of high volumes of solvents, are time consuming, and in some cases have low recoveries [90].

## 6. Conclusions

The use of food colorants in the production of foods leads to the need for the development of accurate, precise, sensitive, and selective analytical methods for their analysis and quantification. Certain interest in the impacts of food colorants is being reported worldwide. There is a plethora of analytical research works that deal with the analytical challenge of the analysis and quantification of either natural or synthetic food colorants. The research community gives more attention to the appropriate analysis, in sufficient concentration or mass fraction levels, mostly to synthetic food colorants rather than natural ones.

Analytical methodologies have much more to offer in this direction and, as it could be concluded from synthetic colorants, HPLC is the most frequently used followed by capillary electrophoresis. In terms of detection methods, the simple UV-Vis/DAD is the predominant one followed by tandem MS. The analytical techniques and sample preparation methodologies presented cover the existing methodologies mainly applied during the last decade.

Regarding sample preparation, this is highly sample dependent. It could involve the application of different extraction techniques, such as membrane filtration, liquid–liquid and solid phase extraction techniques, for cleaning-up the highly complex matrix of food products. Sample preparation is of great importance and must be carefully developed, in order to avoid or eliminate existing matrix interferences aiming to the development of simple, selective, and precise methods of extraction.

In the case of simple liquid samples, dilution and injection are preferred, though in other cases such as high protein content foods, specific steps need to be followed for sufficient sample clean-up.

## Figures and Tables

**Table 1:** Methods for the analysis of natural food colorants in various food products.

Food Colorant	Food Matrix	Analytical Technique	Detection	Detection Settings (i.e., λ, Ionisation)	Column	Elution	Mobile Phase	Inj. Volume	Figures of Merit (LOD, LOQ, Linear Range)	Ref.
3-Deoxy-anthocyanidins	*Sorghum bicolor* (L.) Moench seeds	High Pressure Liquid Chromato-graphy (HPLC)	Diode Array Detection (DAD)	485 nm	Luna C18 column (150 × 4.6 mm, 5 mm)	Gradient	4% HCOOH in H_2_O (*v*/*v*) (Solvent A) and acetonitrile (Solvent B)	20 μL	n/a	[34]
Anthocyanin-derived extracts	*Acacia decurrens* Willd. Bark	Spectrophotometric analysis	UV-Vis	400–800 nm	n/a	n/a	n/a		n/a	[35]
	-*Tulipa gesneriana* L.	Spectrophotometric analysis	UV-Vis	765 nm	n/a	n/a	n/a	n/a	n/a	[36]
Cyanidin 3-glucoside	-*Pistacia lentiscus* L. fruits; -*Santalum album* L. fruits	HPLC	DAD	520 nm, 440 nm, 310 nm and 280 nm	SS Wakosil C18 (150 × 4.6 mm, 5 μm)	Gradient	0.1% trifluoroacetic acid (TFA) in H_2_O (solvent A) and 0.1% TFA in acetonitrile (Solvent B)	20 μL	n/a	[37]
Cyanidin 3-glucoside	-*Pistacia lentiscus* L. fruits; -*Santalum album* L. fruits	HPLC	ESI-MS		SS Wakosil C18 (150 × 4.6 mm, 5 μm)	Gradient	0.1% TFA in H_2_O (solvent A) and 0.1% TFA in acetonitrile (Solvent B	20 μL	n/a	[37]
Betacyanins	-Hylocereus polyrhizus	HPLC	MS	ESI (+)	AQUA C18-reversed phase column, 5 μm	Gradient	(A) 2% (*v*/*v*) CH_3_COOH in H_2_O and (B)0.5% CH_3_COOH in H_2_O/acetonitrile (50/50, *v*/*v*)		n/a	[38]
Betalains	*Beta vulgaris* L. roots	HPLC	UV-Vis	538 nm; 480 nm	Lichrocart 250 × 4 RP-18 (5 μm)	Gradient	H_2_O (A) and acetonitrile (B).	20 μL	n/a	[39]
Betalains	*Opuntia ficus-indica* [L.]	HPLC	UV-Vis	245 nm	Luna C18(2) column (250 × 4.6 mm, 5 µm)	Isocratic	20 mM KH_2_PO_4_/ Acetonitrile 95:5 *v*/*v*	20 μL	n/a	[40]
Betalains	*Opuntia ficus-indica* [L.]	Spectrophotometric analysis	UV-Vis	*λ* = 536 nm	n/a	n/a	n/a	n/a	n/a	[41]
Betalains	*Rivina humilis* L. fruits, juice	Spectrophotometric analysis	UV-Vis	*λ* = 535 nm	n/a	n/a	n/a	n/a	n/a	[42]
α-carotene	*Daucus carota* L. roots	HPLC	UV-Vis	450 nm	Supelcosil LC-18 column (15 cm × 4.6 cm, 5 μm)	Isocratic	Methanol/10% (*v*/*v*) Acetonitrile: H_2_O	50 μL	n/a	[43]
Lutein	Commercial/Milk	HPLC	UV-Vis	450 nm	RP C30 YMC (250 × 4.6 mm, 5 μm)	Isocratic	ethanol, tert-butyl-methyl-ether (MTBE) as the mobile phase	50 μL	n/a	[44]
Lutein	Hawaii “T. erecta”, Carmen “T. patula”.	HPLC	DAD	450 nm	Waters-Spherisorb column SC-04 (125 × 4.0 mm, ODS2, 3.0 μm)	Gradient	(A) Acetonitrile–methanol (9:1 *v*/*v*): (B) Ethyl acetate	100 μL	n/a	[45]
Astaxanthin	Microalgae and yeasts	HPLC	DAD	470 nm	Chiralcel OD-RH column (5 μm, 150 mm × 4.6 mm)		(A) Acetonitrile and (B) phosphoric acid (3.5 mM)	n/a	n/a	[46]
Crocetin, Crocin	Grape seed, monascus, gardenia, and red radish	Spectrophotometric analysis	UV-Vis	438 nm; 462 nm	n/a	n/a	n/a	n/a	n/a	[47]
Monascus red pigments	Beetroot red and paprika extract	High Resolution Mass Spectrometry	HPLC-QTOF-MS	ESI (+)	Kinetex c18 column (2.6 μm, 50 mm × 4.6 mm)	Gradient	(A) Acetonitrile; (B) H_2_O; (C) aqueous HCOOH 1% *v*/*v*	n/a	n/a	[48]

**Table 2:** Sample preparation techniques for the analysis of natural food colorants in food products.

Food Colorant	Extraction/Sample Preparation	Ref.
3-Deoxyanthocyanidins	Ground sample, with 1% HCl in methanol, centrifugation hydrolysis;	[34]
Anthocyanin-derived extracts	Comparison of different extraction methods (ultrasonic and natural extraction) vs. magnetic stirring	[35]
	Extraction with ethanol: H_2_O (1:1 *v*/*v*) acidified with 0.01% HCl	[36]
Cyanidin 3-glucoside	Extraction with 0.1% HCl (*v*/*v*) in methanol, combination of the extracts, evaporation, and dissolution	[37]
Betacyanins	Mixing with water, filtration, addition of ethanol (precipitation of pectic substances and proteins)	[38]
Betalains	Sample dissolution in ethanol, agitated and homogenized	[39]
Betalains	Filtration of water extract (no pH adjustment)	[40]
Betalains	Lyophilization and macerated with PBS (pH 5.0) in 1:5 *w*/*w* ratio, followed by spray-drying	[41]
Betalains	Dilution of the juice; filtration; addition of Se^4+^, Zn^2+^, and Cu^2+^	[42]
α-carotene	Comparison between simple extraction and Supercritical Fluid extraction (CO_2_); Simple extraction: Hexane/acetone; SFE: SC-CO_2_ (SFE)	[43]
Lutein	Sample dilution in 95% ethanol and extraction with acetone and petroleum ether. Evaporation and reconstitution	[44]
Lutein	Extraction with organic solvent (isopropanol), centrifugation and supernatant extracted with hexane	[45]
Astaxanthin	Extraction with ethyl acetate, filtration	[46]
Crocetin, Crocin	Dilution in DMSO	[47]

**Table 3:** Analytical techniques for the determination of natural food colorants in food samples.

Food Colorant	MATRIX	Analytical Technique	Detection	Column	Elution	Mobile Phase	Inj. Volume	Figures of Merit (LOD, LOQ)	Ref.
Brilliant blue	Liquid foods	CE	UV (λ = 220 nm) 36 cm capillary; Separation voltage (8 kV, −8 kV);	Fused-silica cappilaries of 375 od μm and 75 μm i.d	-	30 mM PBS buffer (pH6), with 0.9 mg/mL dASNPs and 2 mM β-cyclodextrin (CD)	Electro kinetic injection	LOD = 0.36 mg/L LOQ = 0.63 mg/L	[2]
Amaranth, ponceau 4R, sunset yellow, tartrazine, brilliant blue		spectrophotometric kinetic method	UV-Vis λ_Prussian blue_ = 760 nm	n/a	n/a	n/a	n/a	LOD = 0.2–6.0 mg/L	[9]
Sudan I	Non-alcoholic drinks, sweets, jellies	Enzyme-linked Immuno-sorbent assay (ELISA)	n/a	n/a	n/a	n/a	n/a	LOD = 0.07 ng/mL	[29]
Sudan I	Non-alcoholic drinks, jellies	HPLC	UV- Vis (478 nm)	C_18_ (250 × 4.6 mm, 5.0 μm)	Gradient	methanol/2% CH_3_COOH	20 μL	LOD = 0.14 ng/mL	[29]
Tartrazine, quinoline Yellow, sunset yellow, Carmoisine, Amaranth, ponceau 4R, Erythrosine, Red 2G, allura Red AC, Patent Blue V, Indigo Carmine, brilliant blue, Green S	Beverages, dairy powders, jellies, candies, condiments, icings, syrups, extracts	HPLC	DAD Various wavelengths	Discovery C18 (250 mm × 4.6 mm 5 μm)	Gradient	CH_3_COONH_4_ 0.13 M (pH = 7.5; NaOH)/methanol: acetonitrile 80:20 v/v	20 μL	LOD = 1.87–22.1 μg/L	[49]
Tartrazine, sunset yellow, brilliant Blue, acid red	Powder	SERS-Raman	confocal microscope Raman spectrometer system	n/a	n/a	n/a	n/a	LOD = 10^−7^ M	[50]
Allura red, sunset yellow, tartrazine	Soft drinks	HPLC	DAD	n/a	Gradient	methanol (HPLC grade) and NaH_2_PO_4_/ Na_2_HPO_4_ buffer (0.10 M, pH = 7.0).	n/a	LOD = 0.06–0.30 μg/mL	[51]
Allura red, sunset yellow, tartrazine	Soft drinks	HLA-Go	-	-	-	-	-	-	[51]
Azorubine, amaranth, cochineal red A, red 2G, allura red, azocarmine B (AZO B), azocarmine G (AZO G), ponceau 2R, ponceau 6R, tartrazine, sunset yellow, quinoline yellow, orange II, metanil yellow (MY), patent blue V, indigo carmine, brilliant blue	Solid food/liquid beverages	HPLC	DAD DAD, λ_quant._: −620 nm (blue); −515 nm (red); −420 and 480 nm (yellow).	C8 (150 × 4.6 mm, 3 μm)	Gradient	Acetonitrile/sodium acetate (pH = 7)	20 μL	5–300 mg/kg (solid food samples) 5–100 mg/L (drinks)	[52]
Brilliant blue, Tartrazine, amaranth, carmine, sunset yellow, allura red, erythrosine	Wine and soft drinks	UFLC	ESI (−)-MS/MS	ODS II (100 mm × 2.0 mm; 2.2 μm)	Gradient	A: Acetonitrile: CH_3_COONH_4_ 5.0 mM/ (B) H_2_O: CH_3_COONH_4_ 5.0 mM	5 μL	LOD = 0.45–1.51 μg/L LOQ = 1.51–5.00 μg/L	[53]
Brilliant blue, tartrazine, amaranth, sunset yellow	Wine and soft drinks	TLC-UV-Vis	UV-Vis	TLC-PET 20 × 20 silica gel	n/a	8 mL 2-propanol and 3 mL NH_4_OH	5 μL (standards) and 30 μL sample	n/a	[54]
Allura red, sunset yellow, tartrazine	Solid food/liquid beverages	Spectrophoto-metric BLLS/RBL	Absorbance spectra-pH data Spectral measurements (300–600 nm) at different pH	n.a	n.a	n.a	n.a	LOD = 0.54 mg/L	[55]
Sunset yellow	Beverage	HPLC	ESI (−)-MS	C18-ether column (150 mm × 4.6 mm, 5 μm)	Isocratic	(A) 63% aqueous solution 20 mM CH_3_COONH_4_: (B) 37% methanol	20 μL	n/a	[56]
Carmoisine, sunset yellow	Beverage	HPLC	ESI (−)-MS	C18 column (250 mm × 2 mm, 4 μm)	Isocratic	(A) Methanol and (B) 10 mM HCOONH_4_ (45:55, *v*/*v*)	20 μL	LOD = 10–12 μg/L	[57]
Allura red	Beverage	HPLC	ESI (−)-MS	HSS-T3 column (2.1 mm 100 mm, 1.8 μm)	Gradient	A: H_2_O: CH_3_COONH_4_ 1.0 mM/ (B) Methanol: CH_3_COONH_4_ 1.0 mM	20 μL	n/a	[58,59]
Brilliant blue, tartrazine, allura red, amaranth, Azorubine, patent Blue V, ponceau 4R	Various food products	HPLC	DAD Various wavelengths	Xterra RP18 column (250 × 4.6 mm, 5 μm)	Gradient	A) 0.1 M CH_3_COONH_4_ in water and (B) 0.1 M CH_3_COONH_4_ in methanol	20 μL	LOD = 0.02–1.49 mg/L	[60]
Brilliant blue, indigo carmine, allura red, carminic acid, ponceau 4R, sunset yellow, tartrazine	Dairy powders, color beverages, jellies, candies, condiments, icings, syrups,	CE	UV (200 nm) Condiiton with 1 M NaOH, H_2_O electrode polarity (25kV)	-	-	Running buffer of pH 10 (20 mM NaOH solution to 15 mM disodium tetraborate (borax) to 20 mM NaOH, until the desired pH	Large-volume injection	LODs 0.05–0.40 μg/mL	[61]
Brilliant blue, indigo carmine, allura red, carminic acid, ponceau 4R, sunset yellow, tartrazine, fast green FCF	Liquid foods	CE	UV (λ = 200 nm) Condiiton with 1 M NaOH, H_2_O electrode polarity (25kV)	-		Running buffer of pH 10 (20 mM NaOH to 15 mM disodium tetraborate (borax) to 20 mM NaOH, until desired pH	Large-volume injection	LOD = 0.002–0.026 μg/mL	[62]
Sunset yellow, carmoisine, amaranth, ponceau 4R, erythrosine, red 2G, allura red	Soft drinks	HPLC	DAD Various wavelengths	Symmetry C18 (Waters, Milford, USA) column (150 mm × 4.6 mm, 5 μm)	Gradient	CH_3_COONH_4_ buffer (1% *w*/*v*) (0.13 M) (pH: 7.5) by addition of 0.1 M aq. NH_3_ (solvent A), methanol (solvent B) and acetonitrile (solvent C)	n/a	0.5–1.4 μg/mL	[63]
Tartrazine, quinoline yellow, sunset yellow, carmoisine, ponceau 4R, allura red, indigo carmine, brilliant blue	Various foods and medicines	HPLC	DAD Various wavelengths	C18 column (250 mm × 4.6 mm, 5 μm)	Isocratic	A) Triton X-100 (0.25%, *v*/*v*) and (B) 50 mmol/L PBS (pH 7)	20 μL	LOD = 0.05–0.44 μg/mL LOQ = 0.05–1.12 μg/mL	[64]
Tartrazine, sunset yellow, azorubine, amaranth, cochineal red, red 2G, allura red AC, Brilliant Black BN, brown FK, Brown HT Patent Blue V, brilliant Blue FCF, Green S	Fish roe	HPLC	DAD Various wavelengths	Xterra RP18 column (250 × 4.6 mm, 5 μm)	Gradient	(a) 100 mmol/L CH_3_COONa buffer (pH 7.0) and (b) Acetonitrile	20 μL	LOD = 0.02--1.49 mg/L	[65]
Brilliant blue, Indigo carmine, allura red, erythrosine, ponceau 4R, sunset yellow, Lemon yellow	Protein-rich samples	HPLC	DAD Various wavelengths	RP-C18 Column	Gradient	Methanol- 20 mM of CH_3_COONH_4_	20 μL	LOD = 0.1–0.4 mg/kg	[66]
Brilliant blue, tartrazine, sunset yellow, amaranth, carminic acid, acid red, allura red	Meat products	UHPLC	PDA Various wavelengths	BEH C18 (100 × 2.1 mm, 1.7 μm	Gradient	Acetonitrile/ CH_3_COONH_4_	5 μL	LOD = 0.01 mg/kg LOQ = 0.05 mg/kg	[67]
Carminic acid, sunset yellow, tartrazine	Non-alcoholic drinks, sweets, jellies	Capillary Electrophoresis (CE)	UV (λ = 220 nm) 36 cm capillary; Separation voltage (8 kV, −8 kV);	Fused-silica capillaries of 375 od μm and 75 μm i.d	-	30 mM PBS buffer (pH6), with 0.9 mg/mL dASNPs and 2 mM β-cyclodextrin (CD)	Electrokinetic injection	LOD = 0.03 -0.072 mg/L LOQ = 0.16–0.31 mg/L	[68]
Amaranth, Ponceau 4R, Sunset yellow, tartrazine, Sudan I-IV	Soft drinks/solid samples	HPLC-ESI (+)-MS	ESI (+)-MS	Spherigel C18 (250 × 4.6 mm, 5 μm)	gradient	aq. methanol 0.1% HCOOH/aqueous methanol 20 mM CH_3_COONH_4_/1% CH_3_COOH	20 μL	LOD = 2.0–3.5 ng LOQ = 5.4–10.5 ng	[69]
Brilliant blue, allura red, amaranth, Erythrosine, ponceau 4R, sunset yellow, tartrazine	Soft drinks and processed meats	HPLC	DAD Various wavelengths	Inertsil ODS-SP column (250 × 4.6 mm, 5 μm)	Gradient	CH_3_COONH_4_ (0.1 M, pH = 7.2)—methanol-acetonitrile (9:1 *v*/*v*)	20 μL	LOD: 0.005 μg/mL LOQ: 0.018 μg/mL	[70]
Brilliant blue, sunset yellow, tartrazine	Dairy powders, beverages, jellies, candies, syrups, extracts	Flow injection (FIA)	Amperometric detection (boron-doped diamondelectrode)	-	-	E_det._2 = −450 mV (100 ms duration) vs. Ag/AgCl (3.0 M KCl).		LOD = 0.8–3.5 μM	[71]
Brilliant blue, sunset yellow, tartrazine	Beverages, jellies, candies, condiments, icings, syrups	Differential pulse voltammetry (DPV)	Cathodically pretreated boron-doped diamond (BDD) elctrode	-	-	30 Hz; amplitude(a), 40 mV; for DPV scan rate (v), 0 mVs	-	LOD = 13.1–143 nM	[72]
Erythrosine, carmoisine, amaranth, ponceau 4R, Red 3G	Syrups	Capillary electrophoresis (CE)	Laser-induced fluorescence detection Various λ_exc._/λ_emis._: (Nd:YAG laser with wavelength 532 nm and power 5 mW)	Fused silica capillary with I.D. 50 μm, O.D. 360 μm, length 30 cm	n/a	V = +17 kV (intensity of electrical field was 460 Vcm^−1^).	n/a	LOD = 0.2–0.4 μg/mL	[73]
Tartrazine, sunset yellow, azorrubine, bordeaux S, ponceau 4R, erytrosine, red no 40, patent blue V, indigo carmin, brilliant blue	Alcoholic beverages	CE	UV/vis PBS 10 mM with sodium dodecyl sulfate 10 mM, pH 11, and +25 kV of voltage	Fused silica capillary (73 cm)	n/a	Phosphate buffer solution of 10 mM with sodium dodecyl sulfate 10 mM, pH 11, and +25 kV voltage	n/a	LOD = 0.4–2.5 μg/mL LOQ = μg/mL	[74]
Tartazine, Amaranth, Sunset yellow, allura red, Lutein, lycopene, β-carotene	Various foodstuff	HPLC	DAD Various wavelengths	C18 column (250 mm × 4.6 mm, 5 μm)	Gradient	1% CH_3_COONH_4_, methanol and acetone;	20 μL	LOD = 0.2–50 ng/mL	[75]
Tartrazine, Quinoline yellow, Sunset Yellow, Carmoisine, Brilliant Blue	Solid foods	spectrophotometric method	UV-Vis Various wavelengths	n/a	n/a	n/a	n/a	LOQ = 1–5 μg/mL	[76]
Allura red	Liquid foods	spectrophotometric method	UV-Vis (506 nm)	n/a	n/a	n/a	n/a	LOD = 2.35 μg/L	[77]
Tartrazine, New red, Amaranth, Ponceau 4R, Sunset yellow, Allura red, Acid red, Brilliant Blue, Acid red, Erythrosine, Acid orange, Basic flavine O, Basic orange, Siperse blue 106, Crystal violet, Leucine malachite green, Leucine crystal violet	Meat	UHPLC	DAD (200–800 nm)	C18 column (2.1 mm × 50 mm, 1.7 μm)	Gradient	(A) 20 mM CH_3_COONH_4_–0.02% acetic acid (pH 5) and (B) acetonitrile	2 μL	LOD = 0.96–2.16 μg/kg LOQ = 1.61–7.19 μg/kg	[78]
New red, Amaranth, Camine, Sunset yellow, Acid Red G, Allura red, Acid Scarlett GR, Erythrosine, Rhodamine B, Sudan I, Para red, Sudan II, Sudan III, Sudan red 7B, Sudab IV, Sudan Orange G	Hotpot condiment	HPLC	DAD Various wavelengths	C18 column (4.6 mm × 250 mm, 5 μm)	Gradient	(A) Methanol; (B) 0.01 M PBS (pH = 7.5)	20 μL	LOD = 0.001–0.00 3 mg/kg	[79]
Allura Red, Ponceau 4R	Granulated drinks	UV–visible spectrophotometer	UV–visible spectrophotometer (ZCDS)	n/a	n/a	n/a	n/a	LOD = 0.059–0.102 μg/mL LOQ = 0.198–0.341 μg/mL	[80]
Brilliant Blue, Sunset Yellow, Tartrazine	Non-alcoholic drinks, sweets, jellies	HPLC	UV-Vis (630 nm, 480 nm, 430 nm)	Modified C18 column (250 × 4.6 mm, 5 μm) with a 0.25% (*v*/*v*) Triton X-100 aq. solution at pH 7	Isocratic	0.25 mL of Triton X-100 (Sigma) up to 100 mL with 50 mmol l−1 phosphate buffer solution at pH 7	20 μL	LOD = 0.143–0.080 mg/L	[81]
Tartrazine, Amaranth, Sunset Yellow, Allura red, Ponceau 4R, Erythrosine	Soft drinks, sugar and gelatin based confectionery	HPLC-UV	UV 430 nm, 510 nm	C18 column (250 mm × 4.6 mm, 5 μm	Gradient	(A) 0.1 mol/L ammonium acetate aqueous solution (pH 7.5, adjusted with 10 mol/L NaOH- methanol–acetonitrile (30:70, *v*/*v*)	20 μL	LOD = 0.015–0.32 ng/mL	[82]
Allurea red, Amaranth, Erythrosine, Ponceau 4R, Sunset Yellow	Beverages, alcoholic drinks and fish foe	SERS-Raman	Radiation of 514.5 nm from an air-cooled argon ion laser was used for SERS excitation	n/a	n/a	n/a	n/a	LOD = 10^−7^–10^−5^ M	[83]
Sunset yellow	beverage, dried bean curd, braised pork	ELISA	n/a	n/a	n/a	n/a		LOD = 25 pg mL^−1^	[84]
(40 food colorants) Ponceau 6R, Tartrazine, Fast yellow AB, Amaranth, Indigotine, Naphthol yellow S, Chrysoine, Ponceau 4R, Sunset yellow FCF, Red 10B, Orange G, Acid violet 7, Brilliant black PN, Allura red AC, Yellow 2G, Red 2G, Uranine, Fast red E, Green S, Ponceau 2R, Azorubine, Orange I, Quinoline yellow, Martius yellow, Ponceau SX, Ponceau 3R, Fast green FCF, Eosine, Brilliant blue FCF, Orange II, Orange RN, Acid blue 1, Erythrosine, Amido black 10B, Acid red 52, Patent blue V, Acid green 9, Phloxine B, Benzyl violet 4B, Rose bengal	Drinks, syrups and candies	HPLC	HPLC–DAD	C18 column (50 mm × 4.6 mm, 1.8 μm)	Gradient	(A) 0.1 mol/L of CH_3_COONH_4_ pH 6.7 and (B) was Methanol–Acetonitrile (50:50, *v*/*v*)	5 μL	LOD = 0.03–0.1 μg/g	[85]
Ponceau 4R, Sunset Yellow, Allura Red, Azophloxine, Ponceauxylidine, Erythrosine, Orange II	Animal feed and meat	LC	ESI (−)-MS	C18 column (2.1 mm × 150 mm, 5 μm)	Gradient	(A) 20 mmol/L CH_3_COONH_4_: Acetonitrile		LOD = 0.02–21.83 ng/mL	[86]
New Coccine, Indigo Carmine, Erythrosine, Tartrazine, Sunset Yellow FCF, Fast Green FCF, Brilliant Blue FCF, Allura Red AC, Amaranth, Dimethyl Yellow, Fast Garnet GBC, Para Red, Sudan I, Sudan II, Sudan III, Sudan IV, Sudan Orange G, Sudan Red 7B, Sudan Red B, Sudan Red G	Chili powders; commercial syrup preserved fruits	LC	ESI (−) and ESI (+) MS/MS	Acclaim Polar Advantage C16 (3 mm, 4.6 × 150 mm)	Gradient	(A) Acetonitrile and (B) 20 mM CH_3_COONH_4_ –1.0% CH_3_COOH		LOQ = 0.005–1 mg/kg	[87]
Multi-class (53 food colorants)	Spices	UHPLC	QTOF-MS (sequential window acquisition of all theoretical fragment-ion spectra- SWATH)	Acquity UPLC BEH C18 column (2.1 × 100 mm, 1.7 μm,)	Gradient	(A) Acetonitrile and (B) 10 mM CH_3_COONH_4_ pH = 6.7	n/a	n/a	[88]
Multi-class (34 water soluble synthetic food colorants)	Beverages, syrup, chewing gum	HPLC	DAD-IT-TOF/MS (λ = 200–700 nm)	Atlantis™ dC18 (4.6 mm × 250 mm, 5 μm)	Gradient	(A)20 mM HCOONH_4_ buffer; (B) methanol/acetonitrile (1:1 *v*/*v*)	20 μL	LODs = 0.009–0.102 μg/mL; LOQs = 0.045–0.203 μg/mL	[89]

**Table 4:** Sample preparation techniques for the determination of synthetic food colorants in food samples.

Food Colorant	MATRIX	Extraction/Sample Preparation	Ref.
Amaranth, Ponceau 4R, Sunset Yellow, Tartrazine and Brilliant Blue		Reduction of iron (III) in sodium acetate/hydrochloric acid solution (pH 1.71) followed by a chromogenic reaction with potassium hexacyanoferrate (III) to form the Prussian blue species	[9]
Sudan I	Non-alcoholic drinks, sweets, jellies	Extraction, sonication, centrifugation, filtration. Sample extracts or liquid samples were filtrated	[29]
Tartrazine, quinoline Yellow, Sunset Yellow, Carmoisine, Amaranth, Ponceau 4R, Erythrosine, Red 2G, Allura Red AC, Patent Blue V, Indigo Carmine, Brilliant Blue FCF, Green S	Dairy powders, color beverages, jellies, candies, condiments, icings, syrups, extracts	Beverages: degassed by stirring Solid: dissolved in water, ultrasonication, filtration	[49]
Tartrazine, Sunset yellow, Brilliant Blue, Acid red	Powder	Fabrication of flower-like silver nanostructures by adding 10 mL of ultrapure water, 2 mL of PVP solution (1%) and 0.2 mL of silver nitrate solution (1 mol/L)	[50]
Allura Red, Sunset Yellow, and Tartrazine	Soft drinks	Food solutions were prepared with dilution with methanol–water mixture (*v*/*v*, 50/50)	[51]
Azorubine, amaranth, cochineal red A, red 2G, allura red, azocarmine B (AZO B), azocarmine G (AZO G), ponceau 2R, ponceau 6R, tartrazine, sunset yellow, quinoline yellow, orange II, metanil yellow (MY), patent blue V, indigo carmine and brilliant blue FCF.	Solid food/liquid beverages	4 g solid sample +20 mL ethanol-H_2_O (1:1 *v*/*v*), ultrasound and shaking, centrifugation, separation, and SPE in polyamide (PA) cartridge Beverages: degas (ultrasound), diluted 1.1 with H_2_O, centrifuge	[52]
Brilliant Blue FCF, Tartrazine, Amaranth, Carmine, Sunset yellow, Allura red, Erythrosine	Wine and soft drinks	Magnetic dispersive solid-phase extraction (M-dSPE):	[53]
Brilliant Blue FCF, Tartrazine, Amaranth, Sunset yellow,	Wine and soft drinks	Degassing followed by SPE with Sep-Pack C18 and elution with 2-propanol	[54]
Allura red, sunset yellow, tartrazine	Solid food/liquid beverages	Sample dilution (0.5–2.0 g) in 100 mL H_2_O	[55]
Sunset yellow	Beverage	No extraction	[56]
Carmoisine, sunset yellow	Beverage	Samples were diluted with water and filtered through 0.2 μm polypropylene membrane	[57]
Allura red	Beverage	filtered through 0.45 μm nylon filter and diluted 1:20 (*v*/*v*) in ultrapure water	[58,59]
Brilliant blue, tartrazine, allura red Amaranth, Azorubine, Patent Blue V, Ponceau 4R	Various food products	Beverages: sample sonicated, addition of aq. NH_3_, filtration; Solid: homogenization, addition of aq. NH_3_, sonication, centrifugation	[60]
Brilliant Blue FCF, Indigo carmine, Allura red, carminic acid, Ponceau 4R, Sunset yellow, tartrazine	Dairy powders, color beverages, jellies, candies, condiments, icings, syrups, extracts	Flavored milk samples: diluted with ethanol (1:1 *v*/*v*), SPE with PA cartridge	[61]
Brilliant Blue FCF, Indigo carmine, Allura red, carminic acid, Ponceau 4R, Sunset yellow, tartrazine, fast green FCF	Liquid foods	Beverages: degas (ultrasound) and directly to CE Milk: diluted with ethanol (1:1 *v*/*v*), SPE with PA cartridge Jelly: blended with ethanol: H_2_O 1:1 *v*/*v* at 65 °C × 4 h + SPE (Polyamide cartridge)	[62]
Sunset yellow, Carmoisine, Amaranth, Ponceau 4R, Erythrosine., Red 2G, Allura red	Soft drinks	Extraction stage, followed by sonification, centrifugation, and concentration step + clean up via SPE on polyamide (PA) cartridges	[63]
Tartrazine, Quinoline Yellow, Sunset Yellow, Carmoisine, Ponceau, 4R, Allura Red, Indigo Carmine, Brilliant Blue	Various foods and medicines	Homogenization, dissolution, filtration	[64]
Tartrazine, Sunset Yellow FCF, Azorubine Amaranth Cochineal Red, Red 2G), Allura Red AC, Brilliant Black BN, Brown FK and Brown HT, Patent Blue V, Brilliant Blue FCF, and Green S	Fish roe	Extraction with aq. NH_3_, centrifugation, pH adjustment, addition of PA sorbent and extraction with methanol	[65]
Brilliant blue, Indigo carmine, allura red, erythrosine, ponceau 4R, sunset yellow, Lemon yellow	Protein-rich samples	Purification/deproteinization with chitosan	[66]
Brilliant blue, tartrazine, sunset yellow, amaranth, carmininic acid, acid red, allura red	Meat products	ASE (static) with ethanol-H_2_O_NH_3_ 75:24:1 *v*/*v*/*v*, 85oC for 10 min	[67]
Carminic acid, sunset yellow, tartrazine, brilliant blue.	Non-alcoholic drinks, sweets, jellies	d-SPME with diamino-moiety functionalized silica nanoparticles (dASNPs) and β-cyclodextrin (β-CD) and pseudo-stationary phases (PSPs). Optimization of pH (2.5), sorbent, amount of dASNPs; ionic strength; extraction time and mode; desorption time; interferences (no interferences identified)	[68]
Amaranth, Ponceau 4R, Sunset yellow, tartrazine, Sudan I-IV	Soft drinks/solid samples	Liquid: filtration, degassing Solid: homogenization, extraction with DMSO, sanitation, centrifugation, and filtration	[69]
Brilliant blue FCF, Allura red, Amaranth, Erythrosine, Ponceau 4R, Sunset Yellow, Tartrazine	Soft drinks and processed meats		[70]
Brilliant blue, sunset yellow, tartrazine	Dairy powders, color beverages, jellies, candies, condiments, icings, syrups, extracts	Homogenization, addition in 0.1 M H_2_SO_4_ (gelatin dissolution), ultrasonic and dilution with supporting electrolyte	[71]
Brilliant blue, sunset yellow, tartrazine	Dairy powders, color beverages, jellies, candies, condiments, icings, syrups, extracts	-	[72]
Erythrosine, carmoisine, amaranth, ponceau 4R, Red 3G	Syrups	Dilution to PBS (20 mM, pH 11) as a background electrolyte (BGE) in the ratio of 1:10 or 1:2 for CE–LIF	[73]
Tartrazine, sunset yellow, azorrubine, bordeaux S, ponceau 4R, erytrosine, red no 40, patent blue V, indigo carmine, brilliant blue FCF	Alcoholic beverages	degassed by mechanical agitation and filtered	[74]
Tartazine, Amaranth, Sunset yellow, Allura red, Lutein, Lycopene, β-carotene	Various foodstuff	Ultrasound-assisted solvent extraction: immersion to methanol, sonication, centrifugation, extraction with acetone, evaporation. Liquid: 0.5 mL + 1 mL methanol; Solid: 0.2 g + 1 mL methanol	[75]
Tartrazine, Quinoline yellow, Sunset Yellow, Carmoisine, Brilliant Blue	Solid foods	Solid: a portion of food was diluted in H_2_O, centrifuges and diluted with equal volume of CH_3_COOH 3 M Liquid: a portion of was diluted in a mixture of NH_3_: ethanol 2:73%, mix, and centrifuged.	[76]
Allura red	Liquid foods	Sample filtered, pH adjusted (4.0), extraction with Acetonitrile by SPE (MCI GEL CHP20P resin)	[77]
Tartrazine, New red, Amaranth, Ponceau 4R, Sunset yellow, Allura red, Acid red, Brilliant Blue, Acid red, Erythrosine, Acid orange, Basic flavine O, Basic orange, Siperse blue 106, Crystal violet, Leucine malachite green, Leucine crystal violet	Meat	Microwave assisted extraction: sample with methanol/H_2_O (95:5 *v*/*v*) followed by SPE (C18 column), evaporation to dryness and reconstitution with methanol	[78]
New red, Amaranth, Carmine, Sunset yellow, Acid Red G, Allura red, Acid Scarlett GR, Erythrosine, Rhodamine B, Sudan I, Para red, Sudan II, Sudan III, Sudan red 7B, Sudab IV, Sudan Orange G	Hotpot condiment	Direct solvent extraction: sample with solvent (acetone-methanol), vortex, centrifugation, evaporation, pH adjustment	[79]
Allura Red, Ponceau 4R	Granulated drinks	Powdered sample dissolved in distilled water,	[80]
Brilliant Blue, Sunset Yellow, Tartrazine	Non-alcoholic drinks, sweets, jellies	Solid: dilution in H_2_O and filtration	[81]
Tartrazine, Amaranth, Sunset Yellow, Allura red, Ponceau 4R, Erythrosine	Soft drinks, sugar and gelatin based confectionery	Ionic liquid dispersive liquid phase microextraction. Sample + ionic liquid [1-Octyl-3-methylimidazolium tetrafluoroborate ([C_8_MIM][BF_4_])] and methanol addition.	[82]
Allura red, Amaranth, Erythrosine, Ponceau 4R, Sunset Yellow	Beverages, alcoholic drinks and fish foe	Synthesis of the G/Ag nanoparticle composite	[83]
Sunset yellow	Beverage, dried bean curd, braised pork		[84]
(40 food colorants) Ponceau 6R, Tartrazine, Fast yellow AB, Amaranth, Indigotine, Naphthol yellow S, Chrysoine, Ponceau 4R, Sunset yellow FCF, Red 10B, Orange G, Acid violet 7, Brilliant black PN, Allura red AC, Yellow 2G, Red 2G, Uranine, Fast red E, Green S, Ponceau 2R, Azorubine, Orange I, Quinoline yellow, Martius yellow, Ponceau SX, Ponceau 3R, Fast green FCF, Eosine, Brilliant blue FCF, Orange II, Orange RN, Acid blue 1, Erythrosine, Amido black 10B, Acid red 52, Patent blue V, Acid green 9, Phloxine B, Benzyl violet 4B, Rose bengal.	Drinks, syrups, and candies	Drinks: degas, evaporation Solid: grind, mixing with solvent and SPE with PA column with 1% NH_3_/ethanol solution	[85]
Ponceau 4R, Sunset Yellow, Allura Red, Azophloxine, Ponceauxylidine, Erythrosine Orange II	Animal feed and meat	Homogenization, extraction with ethanol:NH_3_:H_2_O (80:1:19 *v*/*v*), evaporation, reconstitution	[86]
New Coccine, Indigo Carmine, Erythrosine, Tartrazine, Sunset Yellow FCF, Fast Green FCF, Brilliant Blue FCF, Allura Red AC, Amaranth, Dimethyl Yellow, Fast Garnet GBC, Para Red, Sudan I, Sudan II, Sudan III, Sudan IV, Sudan Orange G, Sudan Red 7B, Sudan Red B, Sudan Red G	Chili powders; commercial syrup preserved fruits	Homogenization and extraction with Acetonitrile twice	[87]
Multi-class (53 food colorants)	Spices	Extraction with H_2_O/methanol/acetonitrile/THF, 9:1:5:5, *v*/*v*/*v*/*v*)	[88]
Multi-class (34 water soluble synthetic food colorants)	Beverages, syrup, chewing gum	Beverages: degassing, pH adjustment, and dilution. Syrup: dilution, sonication, and pH adjustment. Chewing gum: washing with water, pH adjustment.	[89]
